# Vohsen: Acute Rheumatism

**Published:** 1883-08

**Authors:** 


### Vohsen: Acute Rheumatism.

Karl Vohsen, in an interesting article, discusses first the many theories as to the etiology of rheumatism, finds most of them untenable or unsupported by any definite facts, but leans rather to the belief that it is an *acute infectious disease*, due probably to micrococci in the blood. The many complications are discussed in relation to this point.

Childhood suffers from all the complications of acute rheumatism which befall adults. Paralysis of the muscles of the eye is the only one the author has not seen in children. But still rheumatism in children has its own characteristics. The severity and duration of the pain is less on the average than in adults, the duration in adults being two or three weeks, and in children five to eighteen days. The complications in children show still greater differences. Chorea, a frequent complication in early years, is exceedingly rare in adults. Heart affections are very much more frequent in children. From his own cases and from the records of many others, the author finds that the heart is affected in nearly fifty per cent. of cases in children. This complication is as apt to occur in mild cases as in severe ones, in fact, some authors think it more frequent in the subacute cases.

The author then analyzes twenty cases which had recently come under his notice. The ages were between nine and fourteen years. Ne deductions as to hereditary influence or sex could be fairly drawn. In nine cases there was endo- or pericarditis. In none of these was the fever at any time above 103.2° F., and in one case there was no fever.

Swelling of the joints was observed in three cases. The pain was severe, but of short duration. In all cases the salicylate of soda proved promptly effective against the affection of the joints, but had no effect on the development of the cardiac complications. These occurred in about half of all the cases. The mitral valves and the pericardium were most frequently attacked. The lighter forms of rheumatism seemed especially to predispose to the heart troubles, making examination of the heart necessary in all cases.

As to why the heart is affected in children more often than in adults, the author can offer no explanation. Anatomy and physiology give us no theories. The noduli, Jacobi's narrowness of the aorta, and other anatomical points of difference between the child's and the adult's heart, have disappeared before the age at which rheumatism is frequent. The author then argues that the best theory to explain it is that rheumatism is an acute infectious disease, and attacks the heart of the child more often on account of the less power of resistance it has. Further, exactly this relation of the heart to acute rheumatism is an argument in favor of its infectious origin. Of forty-five cases of endocarditis in childhood, reported by v. Dusch, fifteen were idiopathic, twenty were connected with acute, two with subacute rheumatism, and the remainder were complications of outspoken infectious diseases—scarlatina, variola, syphilis congenita. The acute rheumatism, therefore, so far as the heart complication is concerned, would seem to class itself with these acute diseases. Further, the rheumatism especially causes heart complications, because its affection especially attacks synovial membranes, and because there is so marked a parallelism between synovial membranes and the endocardium. All the darkness which surrounds the development and course of acute rheumatism is not removed by the supposition of a specific virus, but the author claims that not only has the theory good facts for its support, but also it explains more of the symptoms and complications than any other theory which has been offered.—*The American Journal of Obstetrics.*

